# Evaluating the Economic and Epidemiological Impact of RSV Hospitalizations in Southern Austria [Southern Austria Respiratory Syncytial Virus INpatient Investigation (ARNI Study)]

**DOI:** 10.1111/irv.70046

**Published:** 2024-11-13

**Authors:** G. Sever Yildiz, E. Resch, V. Strenger, E. Eber, B. Resch

**Affiliations:** ^1^ Research Unit for Neonatal Infectious Diseases and Epidemiology Medical University of Graz Graz Austria; ^2^ Department of Paediatrics and Adolescent Medicine, Emergency Room Medical University of Graz Graz Austria; ^3^ Department of Paediatrics and Adolescent Medicine, Division of General Paediatrics Medical University of Graz Graz Austria; ^4^ Department of Paediatrics and Adolescent Medicine, Division of Paediatric Pulmonology and Allergology Medical University of Graz Graz Austria; ^5^ Department of Paediatrics and Adolescent Medicine, Division of Neonatology Medical University of Graz Graz Austria

**Keywords:** bronchiolitis, economic burden of disease, epidemiology, hospitalization cost, preterm infant, respiratory syncytial virus

## Abstract

**Objective:**

RSV bronchiolitis is a leading cause of hospitalization in infants and young children. We aimed to document the economic burden and epidemiology of RSV over seven seasons in Southern Austria.

**Patients and Methods:**

All RSV‐associated hospitalized (PCR‐proven) children ≤ 5 years of age between 1 October 2015 and 30 April 2022 were collected retrospectively. Demographic and epidemiologic data, along with hospitalization costs (direct and indirect), were calculated.

**Results:**

Among 976 children hospitalized due to RSV infection, 87% were healthy term infants, and 79% were < 12 months old. Prematurity (13%) and pre‐existing conditions (11%) significantly impacted older children—59% of cases in the 2nd compared with 68% in the 1st year of live. RSV‐related hospital costs were approximately €2.0 millions per year (of a total of 60 millions per year). RSV accounted for 19% of hospitalizations due to acute respiratory illness (ARI) in children ≤ 5 years, 37% of all ARI < 6 months, 28% of all ARI < 12 and 6.3% of all‐cause hospitalizations < 12 months of age, respectively.

**Conclusions:**

Every 5th hospitalization due to respiratory illness in children ≤ 5 years of age was associated with RSV, representing 7.9% of all hospitalizations and 3.3% of all paediatric hospitalization costs.

## 1 Introduction

In 2019, it was estimated that worldwide, there were 33 million RSV‐associated acute lower respiratory infection (ALRI) episodes, 36 million RSV‐associated ALRI hospital admissions, 26,300 RSV‐associated ALRI in‐hospital deaths and 101,400 RSV‐attributable overall deaths in children aged 0–60 months [1]. Infants at risk for severe RSV associated lower respiratory tract infection (LRTI) include preterm infants with or without bronchopulmonary dysplasia (BPD), infants and children with haemodynamically significant congenital heart disease (hsCHD), and those with chronic lung disease (CLD), immune deficiency syndrome, immunosuppression (malignancies) or neuromuscular impairment. Hospitalization rates up to 10 times higher compared with healthy term infants have been reported [2, 3]. Despite limited antigenic variation, RSV immunity is short and recurrent infections occur life‐long with the first episode being the most severe one (first RSV season) [4]. RSV is a seasonal virus with infection rates peaking during the cold season in temperate and rainy seasons in tropical climates. In Austria, RSV‐related hospitalizations occur between November and April and most often peak in January and February [5]. This was consistent before the dramatic changes by the COVID pandemic. Viral co‐infections (most common influenza virus, adenovirus and rhinovirus) were diagnosed in 37 cases (5%) during the pre‐pandemic period, resulting in a more severe course of disease. The main risk factors of co‐infection were siblings and crowding. Mortality was 0.27% (2/745), and the two deaths occurred in adolescents co‐infected with influenza A virus and were multi‐handicapped (15 and 20 years of age, respectively) [5].

There are limited data on the burden of RSV disease focussing on the economic impact in Europe [6, 7, 8, 9]. Scarce data are available in Austria regarding health care expenditure associated with RSV‐LRTI hospitalizations based on direct and indirect medical costs. Due to the development of long‐lasting monoclonal antibodies that can be administered only once per season, current data is needed. The aim of this study was to document the burden of disease and to calculate direct and indirect medical costs associated with RSV hospitalizations (health care utilization) by means of a retrospective cohort study.

## 2 Materials and Methods

Retrospectively, all infants and children up to 5 years of age diagnosed with RSV infection and hospitalized between 1 October 2015 and 30 April 2022, at the Department of Paediatrics and Adolescent Medicine of the Medical University Graz, a tertiary care centre in Southern Austria, were included for analysis. Inclusion criteria comprised hospitalization due to respiratory symptoms caused by RSV infection, confirmed by a positive PCR test and documented in the medical chart. Exclusion criteria encompassed cases where RSV was not detected or hospitalizations without respiratory symptoms. Nosocomial RSV infections were also excluded. The study received approval from the local ethic committee (Medical University of Graz, protocol number 34‐971 ex 21/22). Case identification involved all RSV‐associated hospitalizations using ICD10 diagnosis codes (J12.1; J21.0; J20.5; B97.4) and, additionally, a full‐text search for ‘RSV’ during the study period. Our University Hospital hosts the largest Paediatric Department in Styria covering 80% of all births in the southeastern region of Austria with the unique situation that all children are re‐hospitalized here.

### 2.1 Epidemiology

We searched for the population‐based incidence of RSV hospitalizations in the first year of life in southeastern Austria. Furthermore, the description of the clinical course [length of stay (LOS), admission to the intensive care unit (ICU), need for respiratory support (any non‐invasive support like high‐flow nasal cannula (HFNC) and invasive mechanical ventilation)] as well as complications (secondary bacterial infections and co‐infections) and treatment strategies (antibiotics, bronchodilators, intravenous fluids, corticosteroids etc.) were assessed. Additionally, we analysed the demographic, seasonal and medical background of RSV hospitalizations (month of birth and month of admission, medical history, living mainly in rural or urban regions and medication including antibiotics, bronchodilators, corticosteroids, use of palivizumab according to the Austrian recommendations for RSV immune prophylaxis) [10]. Clinical severity of respiratory infection was measured by the modified lower respiratory tract infection (LRI) score [11]. In brief, the LRI score ranges from 0 (*no respiratory infection*) to 5 (*respiratory infection with need of mechanical ventilation*) [11]. Supplemental oxygen was given routinely when oxygen saturation was < 90% measured via pulse oximetry. Data were collected from the local electronic databases openMEDOCS (KAGes, Austria) and medical charts. The typical RSV season was defined from 1 October until 30 April. Criteria regarding the need for hospitalization do not exist and was at the discretion of the attending physician, although in practice, oxygen saturation below 90% often indicated the need for admission. We compared the clinical courses of RSV hospitalizations between the 1st and 2nd year of life and calculated the age‐stratified health care utilization by months of age: 0–< 3, 3–< 6, 6–< 12, 12–< 24 and 24–60. Finally, we calculated the proportion of RSV‐related hospitalizations to all hospitalizations due to acute respiratory tract infection (ARI) (divided among the respective groups in months) and to all hospitalizations within the 1st year of life due to any diseases.

### 2.2 Economic Analysis: Inpatient Cost

Hospital expenditures presented in this study were sourced from the financial and patient management department of the University Hospital in Graz. These figures covered LOS, daily expenses in regular wards and intensive care units, and all medical and nursing costs, including meals. Outpatient clinic costs (emergency room) were obtained from the outpatient clinic fee regulations of the hospital.

Additionally, a comprehensive analysis of expenses related to caregivers or family members staying in the hospital was conducted. These charges varied based on the child's age and the type of accommodation chosen, significantly impacting the overall cost. Accompanying persons' fees per day in the mother–child unit were €7.90 from age of 3 months to < 6 years, €14.50 from 6 to < 10 years, €21.10 from 10 to < 14 years, and €27.70 from 14 years onwards. Co‐placement in a folding bed incurred fees of €5.90 from age of 3 to < 6 years, €10.50 from 6 to < 10 years, €15.10 from 10 to < 14 years, and €19.70 from 14 years onwards.

### 2.3 Economic Analysis: Outpatient Costs

Costs related to private medical practices and transportation were collected from the ‘Institute of Pharmacoeconomic Research’ in Vienna (ipf‐ac.at. Accessed 16 May 2024; https://ipf‐ac.at/en/about‐us/institute) and the website of ‘ÖGK‐Styria’ (Austrian Health Insurance Company Styria), Österreichische Gesundheitskasse.at (published 9 June 2021; accessed 16 May 2024; https://www.gesundheitskasse.at), see references list. These expenses included specialist consultation fees per quarter in Styria, with each quarter representing a three‐month period. Details on transportation costs were obtained through telephone correspondence with ÖGK‐Styria. Family care costs were not included in this cost analysis (loss of productivity at the workplace, transportation costs with a private car or public transport).

Statistical analyses were done using Excel (Microsoft Office, Excel 2013) and SPSS (IBM SPSS Statistics 24). For categorical data, chi‐square or Fisher's Exact tests, and for numerical data, *t*‐test or Mann–Whitney *U* test were used as appropriate. Normality assumption was checked using the Shapiro–Wilk test. Statistical significance was set at *p* < 0.05.

## 3 Results

A total of 976 infants and children were hospitalized due to RSV infection during the study period. Hospitalization data are shown in Table 1.

**TABLE 1:** Hospitalization and treatment data of 976 infants and children < 5 years of age hospitalized between 2015 and 2022 at the Department of Paediatrics and Adolescent Medicine of the Medical University of Graz, Austria.

Parameter	Data
Age at admission (months)	3.8 (0.1–60)
Sex (male: female)	556:420 (57:43)
LRI‐score (*n* = 976)	4 (1–5)
Duration of hospitalization (days)	4.9 (0.1–120)
PICU admission	130 (13)
Length of stay at PICU (days)	6 (1–117)
Supplemental oxygen	546 (55)
Duration of supplemental oxygen (days)	3 (1–34)
Respiratory support^a^	130 (13)
Duration of respiratory support (days)	5 (1–29)
Preterm birth	128 (13)
Underlying conditions	110 (11)
Complications	29 (3)
Deaths (due to RSV)	3 (0.3)
Viral co‐infections	83 (8)
Bacterial co‐infections	138 (14)
Treatment	
Antibiotics	191 (20)
Bronchodilators	590 (60)
Corticosteroids	470 (48)
Nose drops	976 (100)
Intravenous fluids	363 (37)
Ipratropium bromide inhalation	106 (11)
Other treatments	141 (14)

*Note:* Data are given as *n* (%) or median (range).

^a^ Any non‐invasive support like high‐flow nasal cannula (HFNC) and invasive mechanical ventilation.

### 3.1 Epidemiology

One hundred twenty‐eight cases (13%) were born prematurely, and the remaining 87% were healthy term infants. 110 (11%) of the total cohort had underlying conditions. The median gestational age was 34.9 weeks. The median age at admission was 3.8 months (data were missing in 14%). Testing for RSV by PCR (real‐time PCR) was available in all cases from nasopharyngeal swabs. The median duration of RSV hospitalization was 4.9 days. Oxygen therapy was required in 546 (55%) cases, and the median duration of supplemental oxygen was 3 days. For further details, see Table 1.

Diagnoses associated with RSV infection and hospitalization were bronchiolitis in 696 infants (80%), pneumonia in 79 cases (8%), upper respiratory tract infection in 46 (5%), rhinitis in 55 (6%) and respiratory insufficiency in 8 (1%) cases.

There were 130 (13%) admissions to the paediatric intensive care unit (PICU). The median length of stay at the PICU was 6 days. All cases (*n* = 130/13%) required respiratory support with the majority needing high‐flow nasal cannula (HFNC), and the median duration on ventilator support was 5 days. The median LRI‐score of all 976 cases was four. Most infants admitted to and ventilated at the PICU were younger than 3 months. The median duration of respiratory support was 5 days in the first year of life and 6 days in the second year; however, 40% of the children had underlying diseases.

Bacterial and viral co‐infections were diagnosed from blood cultures or PCR tests from nasopharyngeal samples (in most cases, but not all) in 221 cases (23%). Viral co‐infections totalled 90 and bacterial co‐infections 152, giving a total of 242, which exceeds the 221 cases due to overlapping co‐infections in some patients. Comparison of 755 infants without co‐infections and 221 with co‐infections regarding clinical characteristics revealed higher LRI scores (median: 3 vs. 4; *p* = 0.038) and higher rates of supplemental oxygen and respiratory support in those with co‐infections. Details regarding pathogens are shown in Table 2.

**TABLE 2:** Viral and bacterial co‐infections in 976 infants and children < 5 years of age hospitalized between 2015 and 2022 at the Department of Paediatrics and Adolescent Medicine of the Medical University of Graz, Austria.

Viral co‐infections		Bacterial co‐infections	
Rhino−/Enterovirus	29	*Staphylococcus aureus*	42
SARS‐CoV‐2	16	*Streptococcus pneumoniae*	25
Influenza	13	*Haemophilus influenzae*	16
Bocavirus	10	*Staphylococcus epidermidis*	15
Parainfluenza	7	*Moraxella catarrhalis*	11
Metapneumovirus	5	Streptococci group A + B	10
Adenovirus	4	*Klebsiella pneumoniae*	5
HHV‐6	2	*Enterococcus faecalis*	5
CMV	2	*Staphylococcus hominis*	5
HSV	1	*Chlamydia pneumoniae*	4
Paraechovirus	1	*Pseudomonas aeruginosa*	4
		*Escherichia coli*	3
		*Mycoplasma pneumoniae*	2
		*Aerococcus viridans*	2
		*Pantoea agglomerans*	2
		Pneumocystis jirovecii	1

A total of 586 cases (60%) were born during the RSV season. One case requiring ECMO likely contributed significantly to hospital costs, and these costs were included in the total cost analysis. The highest hospitalization rate was found among children born in October (14%), and rates were higher when children were born during the first half of the season compared with the following months, see Table 3.

**TABLE 3:** Influence of month of birth on RSV hospitalization rates in 976 cases < 5 years from the Medical University of Graz 2015–2021.

Month of birth	Relative incidence
January	11.73%
February	5.41%
March	3.35%
April	4.38%
May	4.38%
June	5.67%
July	7.47%
August	9.66%
September	12.63%
October	14.05%
November	10.18%
December	11.08%

**FIGURE 1:**
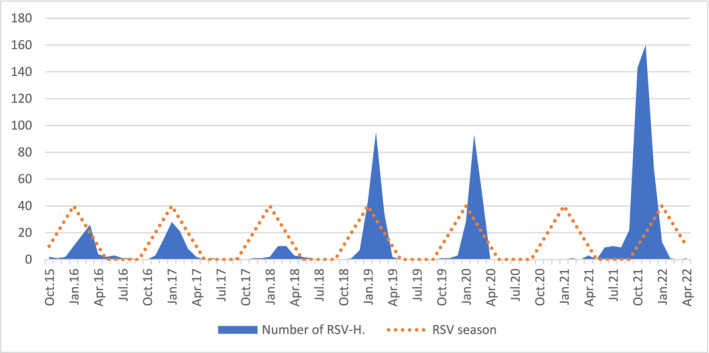
Seasonal distribution of 976 RSV hospitalizations from October 2015 to April 2022 at the Medical University of Graz, Austria. RSV‐H = RSV hospitalizations.

The seasonal distribution of RSV hospitalizations is shown in Figure 1, demonstrating the main season lasting from October to March. The highest hospitalization rate occurred in February (239 children). Most hospitalizations (425 cases) associated with RSV infection were observed during the seasons 2020/2021 and 2021/2022 following the end of the COVID‐19 lockdown. The incidence of RSV hospitalizations was 1.35 in urban and 0.65 in rural regions, respectively, per 1000 inhabitants.

Risk factors for hospitalization and severe RSV infection were seen in 238 (25%) cases. The main risk factor was prematurity with 128/976 cases (13%). A haemodynamically significant CHD was diagnosed in 44/976 (4.5%) cases and neuromuscular diseases in 29 (3%) cases. Other risk factors included chronic lung diseases (cystic fibrosis, asthma bronchiale and primary ciliary dyskinesia) in 14 (1.4%) cases; immune deficiency syndrome or malignancies in 11 (1.1%); and Down syndrome in 7 (0.7%) and bronchopulmonary dysplasia in 5 (0.5%) cases. Comparison of underlying conditions and LRI scores revealed 68% of hospitalized children up to 12 months of age with an LRI score of 4 to 5 had underlying conditions compared with 59% of children between 12 and 24 months of age.

Most hospitalized infants (*n* = 776, 79%) were below 12 months of age. Ninety‐six (10%) cases ranged between 12 months and up to 24 months, 50 (5%) were between 24 and up to 36, 26 (3%) were between 36 and up to 48, and 28 (3%) were between 48 and 60 months, respectively.

**FIGURE 2:**
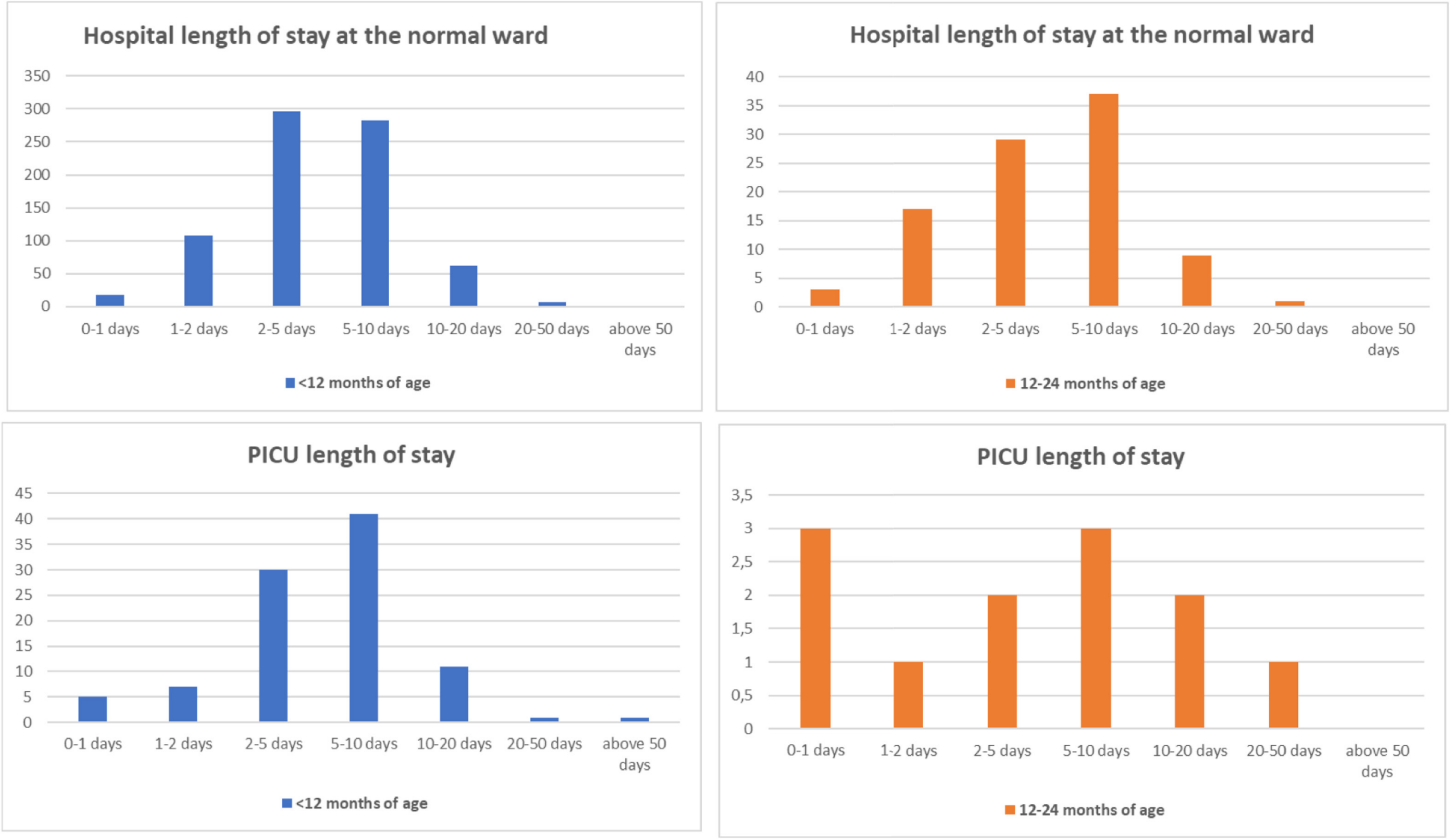
Comparison of length of stay (RSV hospitalizations) at the standard ward and the paediatric intensive care unit (PICU) between first and second year of life between October 2015 and April 2022 at the Medical University of Graz. PICU = paediatric intensive care unit.

There was no difference in the clinical course of RSV hospitalizations between children at 1 and 2 years of age. Children admitted after 12 months of age had the same severe course as children under 12 months of age. The need for oxygen supplementation was even higher in children over 12 months of age. Fifty‐nine per cent of infants had underlying diseases and/or a history of prematurity. Even in ventilated children, we observed no difference between 1 and 2 years of age, see Figure 2. The median duration of hospitalization was 5 days in the majority of all age groups, and lower in infants between 3 and 6 months of age (median: 3.8 days). The need for oxygen supplementation was also similar for all age groups except for children between 3 and 6 months of age. This age group had the lowest rate of underlying diseases, see Table 4.

**TABLE 4:** Comparison of the 976 RSV‐related hospitalizations by age group.

Parameter	0–< 3 months	3–< 6 months	6–< 12 months	12–< 24 months	24–60 months
Number of cases	402 (41.2)	238 (24.4)	136 (13.9)	94 (9.6)	106 (10.9)
Age at admission	1.6 (0.1–3)	4.1 (3–6)	7.9 (6–12)	16.6(12–24)	36.5(24–60)
Length of stay	5.5 (0.5–120)	3.8(0,6–47)	5 (0.7–39)	5 (0.8–35)	5 (0.1–22.5)
LRI score	4 (1–5)	3 (1–5)	4 (1–5)	4 (1–5)	4 (1–5)
Oxygen supplementation	247 (61)	92 (39)	79 (58)	58 (62)	68 (64)
Duration of oxygen supplementation	3 (1–12)	6,2 (1–14)	4 (1–34)	4 (1–12)	3 (1–13)
PICU admission	72 (30)	12 (5)	11 (8)	12 (13)	20 (19)
Length of stay at the PICU	5.2 (0.1–117)	6.2 (1–14)	6.5 (2–26)	6 (0.9–26)	5 (1–14)
Respiratory support	72 (30)	12 (5)	11 (8)	12 (13)	20 (19)
Duration of respiratory support	5 (0.5–18)	6 (1–29)	5 (1–25)	7.5 (0.9–24)	5 (1–13)
Underlying diseases (110)	19 (21)	12 (13)	20 (22)	17 (19)	42 (46)
Prematurity (128)	47 (54)	27 (35)	22 (28)	20 (26)	17 (22)

*Note:* Data are given as median (range) or *n* (%).

The rate of RSV infections of all acute lower respiratory tract illnesses was in total 976 of 5211 hospitalized cases (19%). According to age groups 0–< 3 months 402/1420 cases (28%), 3–< 6 months 238/635 cases (37%), 6–< 12 months 136/689 (20%), 12–< 24 months 94/973(9.7%) and 24–60 months 106/1494 (7%).

We found RSV related hospitalizations being 7.9% (976 out of 12,392) of all‐cause hospitalizations during the study period.

### 3.2 Economic Analyses

RSV‐related hospital costs for hospitalized children were about €2 million per year (1 day of standard care equals €1412.85), of which 60% accounted for standard care and 40% for intensive care (1 day intensive care equals €4716.50). Table 5 shows the emergency room costs at the paediatric department for all hospitalized cases, and Table 6 show the outpatient costs for hospitalized cases. Standard ward costs during the study period were about €8,020,749 (per year €1,233,961). PICU costs amounted to €4,518,407 (per year €695,140). Thus, the total costs amounted to €1,929,101 per year.

**TABLE 5:** Costs associated with emergency room visits for the hospitalized patients (given in €).

Cost factor	Single costs	Study costs (*n* = 976)
Initial examination	34.70	33,867.20
Medical report	105.00	102,480.00
RSV PCR test	126.49	123,454.24
Blood tests	25.10	24,497.60
Conventional X‐ray	70.00	68,320.00
Nasal care (suctioning and nose drops)	14.40	14,054.40
Inhalation therapy (salbutamol)	14.40	12,484.80
Inhalation therapy (ipratropium bromide)	14.40	1526.40
Intravenous line	49.90	18,113.70
Fluid bolus	14.40	12,484.80
Antipyretics	14.40	1526.40
Supplemental oxygen	14.40	7027.20
	Sum	419,845.20

**TABLE 6:** Selected inpatient and outpatient costs (given in €) according to the Austrian Health Insurance Fund of Styria as of 1 January 2019.

	Single costs	Study costs^c^
Cost factor (inpatient)		
Accompanying persons^a^	20.00	19,520.00
Mother–child unit^a^	9.43	1000.00
Folding bed^a^	5.90	625.40
Cost factor (outpatient)		
Medical consultations (1st/quarter)	20.00	1520.00
Subsequent consultations	6.94	4880.00
Weekday visit	26.76	13,058.88
Urgent visit	35.73	17,436.24
Transportation (sitting/lying)	30.00/60.00	43,920.00
Loss of productivity in case of death^b^	2,663,529.40	7,990,558.20

^a^ In case of children > 3 years.

^b^ Human capital approach.

^c^ For all hospitalized patients prior to admission.

The costs of hospitalization together with inpatient and outpatient costs and loss‐of‐productivity costs in case of death revealed approximately €3.5 million per year. The all‐cause hospitalization costs of the paediatric department were estimated to 60 million per year (for 2021); thus, RSV constituted 3.3% of the yearly hospitalization costs (excluding loss‐of‐productivity costs in case of death).

## 4 Discussion

Our study provides a comprehensive analysis of the economic and epidemiological burden of RSV infection in infants and children up to the age of 5 years, particularly focusing on hospitalizations and associated costs in Southern Austria over a 7‐year period. RSV is a significant cause of respiratory illness in children worldwide, particularly in infants, and understanding the epidemiology, clinical course and economic impact is crucial for healthcare planning and resource allocation [7, 8, 9, 12, 13, 14, 15, 16, 17, 18, 19]. This economic analysis provides insights into the direct and indirect medical costs associated with RSV hospitalizations, including hospital stays, emergency room visits, specialist consultations, transportation and caregiver expenses. The substantial financial burden estimated in the study underscores the importance of preventive measures, such as RSV immunoprophylaxis, particularly in high‐risk populations. Moyes et al. [17] incorporated a broader assessment in South Africa of both direct and indirect costs, including caregiver productivity losses and out‐of‐pocket expenses. To our knowledge, there is no other comparable study in Austria. The costs for RSV hospitalizations are about 3.5 million, including direct and indirect costs. It is crucial to acknowledge that as care costs become a substantial component of the overall expenses linked to RSV hospitalizations, it is imperative to account for care expenses during parental leave, along with the ensuing productivity loss in the workplace. Furthermore, transportation costs to the hospital, whether by private car or public transportation, should be incorporated. While our study provides a detailed analysis of the direct medical costs associated with RSV hospitalizations in Southern Austria, it is valuable to contextualize our findings with studies examining economic implications in other regions. It is likely that our cost estimates during the pandemic are underestimated due to fewer RSV‐related hospitalizations in those periods. Demont et al. [6] reported average costs ranging from €4000 to €5000 per RSV hospitalization in France. Our study's per‐day costs for standard care (€1412.85) and intensive care (€4716.50) align closely with Demont et al.'s average hospitalization costs, underscoring the high financial burden of RSV hospitalizations across different healthcare settings [6].

The study of Mao et al. [7] estimated significantly less mean costs per RSV episode at €399.5 (healthcare payer perspective) and €494.3 (societal perspective) [7]. Greenough et al. [8] estimated median cost of £2630 (approx. €4000, US$4800) for RSV hospitalized preterm infants with CLD compared with the total costs of €41.9845 in our study, underscoring the differential financial impact of RSV‐related hospitalizations across specific populations and healthcare settings [8].

Our study can be contrasted with the study of Martinson‐Torres et al. [9] who demonstrated a comprehensive assessment of the burden of medically attended acute lower respiratory infection (ALRI) cases potentially attributable to RSV in Spanish children. The study of Martinos‐Torres et al. [9] found mean direct healthcare costs per medically attended RSV‐ALRI case of €1.753 in the first year of life, €896 in the second, and €683 between 2 and 5 years old. Our analysis revealed higher costs in RSV‐related cases, particularly due to hospitalization: €3362 in the first year of life (72.9% from hospitalizations); €3252 in the second year of life (72.1%), and €3514 in children between 2 and 5 years old (74.2%). This underscores the need for improved RSV testing and codification and the adoption of preventive measures to safeguard all infants, especially during their first year of life.

In comparison to the estimated mean annual cost ($137 billions) of RSV‐associated illness in children aged < 5 years as reported by Moyes et al. [17], our study offers a more detailed breakdown of cost distribution. While Moyes et al. [17] provided an overall cost estimate, our analysis likely provides a more granular perspective, delineating costs across different categories. For instance, our study may have elucidated the proportion of costs incurred by the healthcare system, out‐of‐pocket expenses and indirect costs. Additionally, our investigation into age‐specific cost distributions sheds light on variations in cost burden across different age groups, providing valuable insights for targeted interventions and resource allocation.

The study by Zhang et al. [18] investigates the global burden of respiratory syncytial virus (RSV) and its economic impact, focusing on both direct healthcare costs and indirect costs such as productivity losses. It highlights the significant financial strain on healthcare systems and families, especially in regions with high RSV prevalence. In addition, Del Riccio et al. [19] conducted a systematic review of RSV epidemiology and economic burden across Europe. This study provides a broader perspective on the economic impact of RSV infections, potentially offering comparative data to contextualize the findings of our study in Southern Austria. The findings in our study corroborate existing knowledge about RSV, indicating that infants, especially those under 6 months of age, are at the highest risk of severe disease requiring hospitalization. Prematurity, congenital heart disease and other underlying conditions increase the susceptibility to severe RSV infection. Nevertheless, the majority of infants below 2 years of age, who were hospitalized due to severe RSV infection, are healthy term infants (82%, 797/976). The study also highlights the seasonality of RSV, with peak hospitalizations occurring during the colder months, which aligns with global patterns. Additionally, the incidence of RSV hospitalizations was calculated, providing valuable population‐based data for understanding disease burden. The clinical course of RSV infection described in the study reflects the spectrum of illness severity, ranging from mild upper respiratory tract symptoms to severe lower respiratory tract disease necessitating intensive care and mechanical ventilation. The need for respiratory support, oxygen supplementation and pharmacological interventions such as bronchodilators and corticosteroids underscore the challenge of managing RSV‐associated respiratory distress in hospitalized children despite the missing evidence for the latter [20, 21]. Our findings again highlight the occurrence of bacterial and viral coinfections, which can exacerbate the severity of illness and complicate clinical management [5].

In comparison with our recent study [5], we observed more than half of the patients being born during the season. Additionally, we observed a longer main RSV season extending from December to April. This discrepancy in the duration of the RSV season suggests potential differences in regional epidemiological patterns, possibly influenced by factors such as climate, the COVID‐19 pandemic, population density and healthcare practices. Understanding such variations is crucial for optimizing preventive measures and resource allocation tailored to specific geographic contexts. In short, compared with our recent findings there were a lot of similarities until.

COVID‐19 and non‐pharmacological interventions changed epidemiologic features. The low mortality rates associated with RSV‐related hospitalizations persisted, with fatal cases often linked to co‐infection with other respiratory viruses and pre‐existing medical conditions [5]. Co‐infections in our study were associated with more severe clinical outcomes, including higher LRI scores and increased need for supplemental oxygen and respiratory support, which likely correlates with higher hospitalization costs; however, we did not explicitly collect cost‐related data in this study. Nonetheless, variations in the characteristics of fatal cases underscore the complexity of RSV‐associated outcomes and the need for further research to elucidate underlying risk factors and mechanisms. Hence, the GOLD RSV research group was very successful regarding these aspects in case of fatal RSV infection. In a study from Spain, a mortality rate of 0.08% for RSV and non‐RSV bronchiolitis with or without co‐morbidities was observed, comparable to our findings [22].

The study's strengths lie in its comprehensive approach, including a large sample size, detailed clinical data collection and economic analysis. However, several limitations should be considered, including its retrospective nature, potential selection bias inherent in single‐centre studies, and reliance on diagnostic coding for case identification, which may underestimate the true burden of RSV disease. The study underscores the need for multifaceted approaches to mitigate the burden of RSV infection, including immunization strategies, promotion of preventive measures such as hand hygiene and respiratory etiquette, and targeted interventions for infants entering their first RSV season. Furthermore, the economic analysis emphasizes the importance of cost‐effective interventions such as RSV immunoprophylaxis with long‐lasting monoclonal antibodies, to reduce healthcare utilization and alleviate the financial strain on healthcare systems and families. Family care costs were not included in this cost analysis (loss of productivity at the workplace, transportation costs with a private car or public transport). This is a limitation as indirect costs are essential components of overall societal expenditure.

## 5 Conclusions

RSV‐related hospital costs were about €2 million per year in Austria. Preterm infants accounted for 13%, cases with underlying conditions for 11% of the study population; hence, 87% were healthy term infants and children. RSV hospitalizations accounted for 19% of all hospitalizations due to respiratory illness in children ≤ 5 years of age and were associated with high morbidity and costs. Prematurity and pre‐existing conditions played a major role in older children resulting in comparably severe courses of disease during the 2nd compared with the 1st year of life.

## Author Contributions

**Sever Yildiz G:** writing – original draft, visualization, validation, methodology, project administration, formal analysis, software, data curation, resources, investigation, conceptualization. **Resch E:** supervision, visualization. **Strenger V:** validation, writing – review and editing. **Eber E:** writing – review and editing, validation. **Resch B:** conceptualization, funding acquisition, writing – original draft, project administration, validation, visualization, writing – review and editing, methodology, formal analysis, supervision.

## Conflicts of Interest

Gülsen Sever Yildiz received honoraria for lectures and congress support from Sanofi and Astra Zeneca. Elisabeth Resch received congress support from Nutricia. Volker Strenger received honoraria for lectures and advisory board meetings from Abbvie, Angelini, Chiesi, Diasorin, GSK, MSD, Nestlé, Pfizer, Sanofi, Shionogi und Vifor. Ernst Eber received honoraria for lectures and advisory board meetings from Chiesi, Gilead, Insmed, Vertex and Vifor. Bernhard Resch received honoraria for lectures and advisory board meetings and support for travel costs for medical congresses from Sanofi, AstraZeneca, MSD, Abbvie, GSK, Pfizer, Germania, Chiesi, Nutricia and Nestlé.

### Peer Review

The peer review history for this article is available at https://www.webofscience.com/api/gateway/wos/peer‐review/10.1111/irv.70046.

## Data Availability

The authors have nothing to report.
